# Characterization of NFE2L1-616, an isoform of nuclear factor-erythroid-2 related transcription factor-1 that activates antioxidant response element-regulated genes

**DOI:** 10.1038/s41598-023-47055-2

**Published:** 2023-11-14

**Authors:** Daniel V. Ho, Kaylen G. Suryajaya, Kaitlyn Manh, Amanda N. Duong, Jefferson Y. Chan

**Affiliations:** Department of Laboratory Medicine and Pathology, University of California, Irvine, D440 Medical Sciences, Irvine, CA 92697 USA

**Keywords:** Molecular biology, Transcription

## Abstract

The NFE2L1 transcription factor (aka Nrf1) is a basic leucine zipper protein that performs a critical role in the cellular stress response pathway. Here, we characterized a novel variant of NFE2L1 referred to as NFE2L1-616. The transcript encoding NFE2L1-616 is derived from an intronic promoter, and it has a distinct first exon than other reported full-length NFE2L1 isoforms. The NFE2L1-616 protein constitutively localizes in the nucleus as it lacks the N-terminal amino acid residues that targets other full-length NFE2L1 isoforms to the endoplasmic reticulum. The expression level of NFE2L1-616 is lower than other NFE2L1 isoforms. It is widely expressed across different cell lines and tissues that were examined. NFE2L1-616 showed strong transcriptional activity driving luciferase reporter expression from a promoter containing antioxidant response element. Together, the results suggest that NFE2L1-616 variant can function as a positive regulator in the transcriptional regulation of NFE2L1 responsive genes.

## Introduction

The NFE2L1 (Nuclear factor erythroid 2 (NF-E2)-elated factor-like-1) gene also known as Nrf1, belongs to the cap‘n’collar subfamily of basic-leucine zipper (bZIP) transcription factors that dimerize with other bZIP proteins such as small MAFs, to activate expression of cytoprotective genes through the antioxidant response element (ARE)^1–5^. Ablation of NFE2L1 in mice leads to embryonic lethality indicating that it is an essential gene^6^. Tissue-specific knockout studies indicate that NFE2L1 protects against neurodegeneration and development of steatohepatitis and liver neoplasia^7,8^. Aside from its role in the cellular stress response, studies suggest that NFE2L1 also plays a role in lipid metabolism, and bone remodeling through regulating the differentiation of osteoblast and osteoclast^9–14^.

The NFE2L1 gene encodes several protein isoforms^15,16^. TCF11 and Nrf1a proteins represent long isoforms of the gene. Newly synthesized TCF11 and Nrf1a resides the endoplasmic reticulum (ER) as type II membrane proteins via an N-terminal domain shared by both isoforms^17^. Nrf1a differs by an internal exon encoding a 30-amino acid peptide found in TCF11^15,16^. In the ER, they are N-glycosylated and to enter the nucleus, TCF11 and Nrf1a undergo retrograde translocation into the cytoplasm, where they are deglycosylated by NGLY1 (N-glycanase 1) and cleaved by DDI2 (DNA damage inducible 1 homolog 2)^18,19^. Under basal condition, little Nrf1a and TCF11 accumulate outside of the ER because of proteasome mediated degradation in the cytoplasm. When proteasome activity is low, Nrf1a and TCF11 escape ubiquitination and degradation leading to their accumulation and import into the nucleus to mediate gene expression^20^. Shorter protein isoforms have also been described, but evidence supporting their expression and function is currently insufficient^21–24^.

In this study, we characterize NFE2L1-616, a novel NFE2L1 variant that is lacking the ER targeting domain present in Nrf1a and TCF11. We describe the molecular properties of NFE2L1-616 and its implications for cellular stress response.

## Results

### Isolation of NFE2L1-616 as a novel isoform of NFE2L1

**Figure 1:**
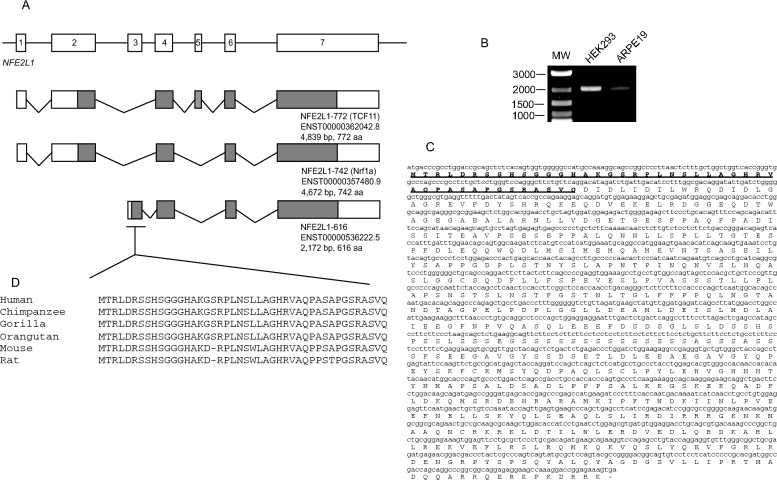
Schematic representation of NFE2L1-616. **(A)** Schematic diagram of human NFE2L1 gene, and transcripts encoding the NFE2L1-772 (TCF11), NFE2L1-742 (Nrf1a) and NFE2L1-616 isoforms. Exons are depicted as boxes and translated portions of transcripts are shown as filled-in regions. **(B)** Agarose gel electrophoresis of RT-PCR amplification products of NFE2L1-616 from HEK293 and ARPE19 cells. (Lane 1) DNA marker. (Lane 2) HEK293. (Lane 3) ARPE19. **(C)** Nucleotide sequence and deduced amino acid sequence of NFE2L1-616. The unique N-terminus (44 amino acids) is bolded and underlined. **(D)** Sequence alignment of the 44 amino acid region of orthologous NFE2L1-616 representatives from various mammals.

The genome organization of various protein coding isoforms encoded by NFE2L1 are depicted in Fig. 1A. TCF11 and Nrf1a are isoforms that have been previously reported and the subject of many investigations into this transcription factor to date. In addition to these two isoforms, the human Ensembl database (GRCh38 release 104) predicts an uncharacterized isoform (ENST00000536222.5) that is supported by multiple ESTs. We propose to name this isoform as NFE2L1-616 (numbering based on amino acids encoded by the isoform), and previously described TCF11 and Nrf1a proteins as NFE2L1-772, and NFE2L1-742, respectively. The NFE2L1-616 transcript is 2172 nucleotide in length encoding a protein of 616 amino acid residues, and it contains a unique N-terminus consisting of 44 amino acids encoded by a distinct 5′ exon not shared with the other NFE2L1 transcripts (Fig. 1A).

To determine whether the ENST00000536222.5 transcript in Ensembl represents a bona fide mRNA, RT-PCR was done to amplify the corresponding full-length cDNA from human DNA. Nested oligonucleotide primers complementary to the unique 5ʹ exon of NFE2L1-616 in combination with primers to the common terminal exon shared by all isoforms were used for PCR. Using mRNA isolated from HEK293 cells, a product of approximately 2 kb was amplified (Fig. 1B). A similar sized amplicon was also detected in ARPE19 cells (Fig. 1B). The identity of these PCR products was confirmed by sequencing which is predicted to result in a novel 44-amino acid sequence at its amino terminus (Fig. 1C). A search of the Ensembl database showed that NFE2L1-616-like isoforms containing the N-terminal 44 amino acid motif are also found in other mammals suggesting that this isoform is conserved in other species (Fig. 1D).

### Alternative exon and promoter of NFE2L1-616

**Figure 2:**
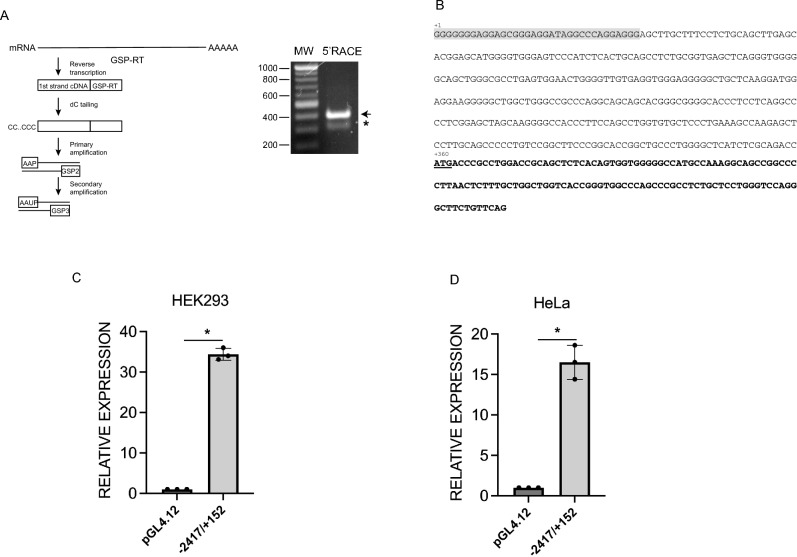
Identification of transcription start site and analysis of promoter activity of the 5′-flanking region of NFE2L1-616. **(A)** Schematic diagram of the 5′-RACE procedure. Reverse transcription was performed on mRNA from HEK293 cells using a NFE2L1 gene specific primer followed by addition of non-templated dCMP residues to the 3ʹ-end of cDNA. Primary and secondary PCR was then carried out using 5ʹ-RACE abridged anchor primers (AAP and AAUP) and gene specific primers (GSP2 and GSP3), and products were resolved on agarose gel. Image shows agarose gel (1%) analysis of the second nested PCR products, which were confirmed by DNA sequencing. The 400 bp NFE2L1-616 5ʹ-RACE product is indicated by an arrow, and a non-specific amplification product is indicated by an Asterix. **(B)** The nucleotide sequence of the 5ʹ′-UTR and portion of the coding region of NFE2L1-616. The start of the transcript is marked as nucleotide + 1, the initiation codon (ATG) is underlined, and coding region is shown in bold letters. Additional nucleotides extending 5ʹ from the annotated exon 1 of NFE2L1-616 are highlighted in gray. **(C)** Promoter activity of the 2 kb region flanking the 5ʹ region of NFE2L1-616 transcript analyzed by luciferase reporter assay in HEK293 and **(D)** HeLa cells. Values are relative to the empty pGL4 vector and represent the mean of three independent experiments each containing 3 replicates, and error bars represent SD, *P < 0.01. P values were calculated by Student’s t-test.

To validate the alternative 1st exon of NFE2L1-616, 5ʹ RACE (Rapid Amplification cDNA Ends) experiment was performed. Messenger RNA isolated from HEK293 cells was reverse-transcribed with gene-specific primers, and cDNA fragments generated were further amplified using a nested PCR scheme (Fig. 2A). As shown in Fig. 2A, a distinct amplicon of approximately 400 bp was detected. Sequencing analysis of the product generated from 5ʹ-RACE confirmed that the transcript extends an additional 51 additional nucleotides in a 5ʹ-direction from the annotated exon 1 of NFE2L1-616, which correlated with the genomic sequence (Fig. 2B). RACE products that continued beyond this sequence were not identified.

To test the assumption that the intronic region located between the second and third exons of NFE2L1-772 and -742 possesses promoter activity, we examined whether 5ʹ flanking region of NFE2L1-616 is transcriptionally active. A 2 kb fragment that flanks the 5ʹ region of NFE2L1-616 transcript was cloned into a promoter-deficient luciferase reporter vector (pGL4-Basic). When transiently transfected into HEK293 and HeLa cells, the genomic region upstream of NFE2L1-616 conferred activation of reporter expression compared to pGL4-Basic vector alone (Fig. 2C,D). These results confirm the promoter functionality of the genomic sequence upstream of exon 1 of NFE2L1-616.

### NFE2L1-616 transcript is expressed in multiple cell lines and tissues

**Figure 3:**
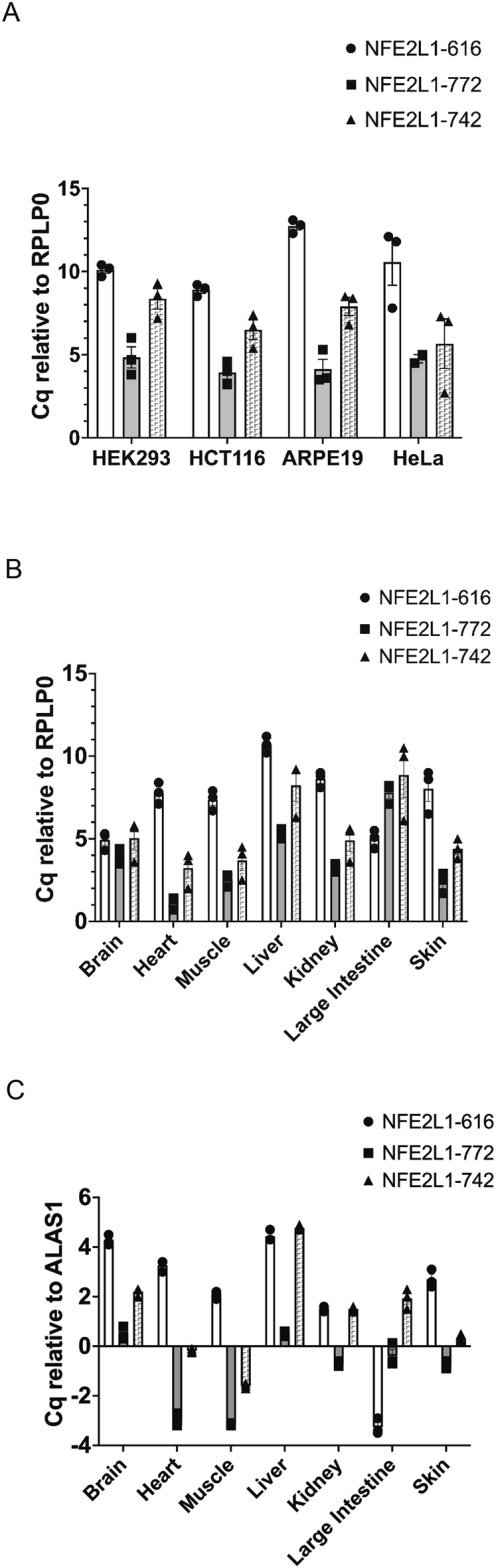
Expression of NFE2L1 isoforms in cells and tissues. **(A)** Real-time PCR analysis of NFE2L1-772, -742 and -616 transcript levels in the indicated cell lines and **(B)** human tissues. Results were calculated using the ΔΔCT method with RPLP0 or ALAS1 as reference genes and represented as Cq (quantification cycle) values for each isoform (mean ± SD of 3 replicates).

To further confirm the presence of NFE2L1-616 transcript, quantitative reverse transcriptase polymerase chain reaction (RT-qPCR) using exon specific Taqman probes was performed. The NFE2L1-616 transcript was detectable in the cell lines that were examined including HEK293, HeLa, HCT116 and ARPE cells (Fig. 3A). In these cell, NFE2L1-616 was expressed at lower abundance in comparison with NFE2L1-772 and NFE2L1-742. NFE2L1-616 transcripts were also detected in adult human tissues including brain, heart, muscle, colon, kidney, skin, and liver (Fig. 3B,C). Using RPLP0 and ALAS1 as reference genes, expression in general was similar compared to NFE2L1-772 and NFE2L1-742, except for colon where NFE2L1-616 is expressed at higher levels to NFE2L1-772 and NFE2L1-742. These results suggest that NFE2L1-616 is expressed ubiquitously.

### Expression and subcellular localization of NFE2L1-616 protein

**Figure 4:**
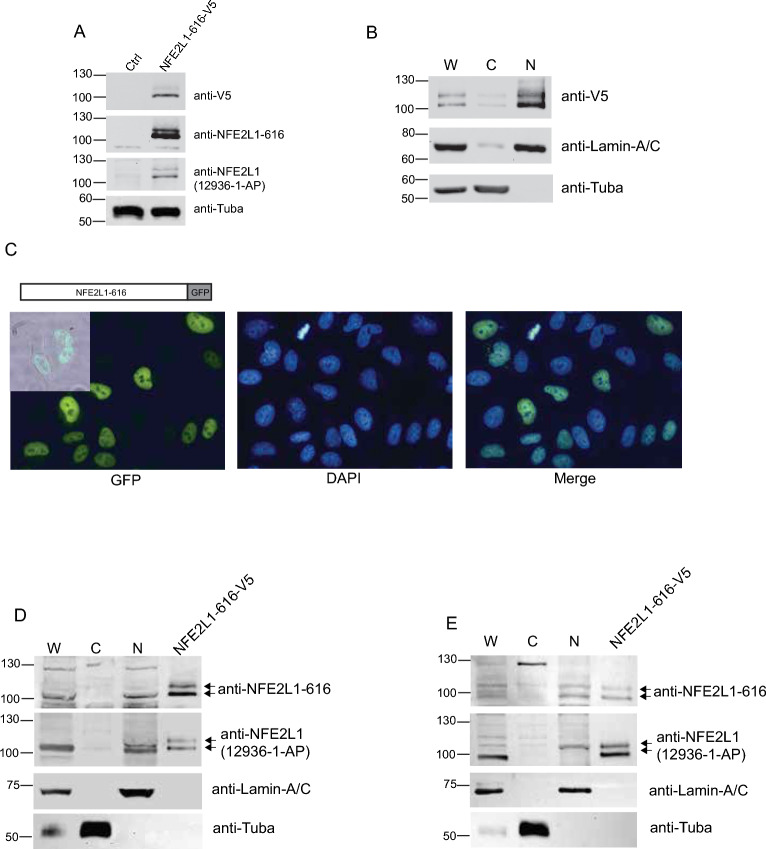
Expression and subcellular distribution of NFE2L1-616 protein. **(A)** Recognition of NFE2L1-616 protein by the isoform specific anti-NFE2L1-616 antibody. Lysates prepared from HEK293 cells expressing V5-epitope tagged NFE2L1-616 were immunoblotted with anti-V5 (top panel), anti-NFE2L1-616 (second panel), and 12936-1-AP (non-isoform specific NFE2L1 antibody, third panel). Loading was controlled by immunoblotting with anti-alpha tubulin antibody (bottom panel). **(B)** V5-epitope tagged NFE2L1-616 is localized to the nucleus. Lysates from HEK293 cells transfected with NFE2L1-616-V5 were fractionated and whole cell lysate (W), cytosolic fraction (C), and nuclear fraction (N) analyzed by SDS-PAGE and western blotting with anti-V5 antibody. Alpha-tubulin and Lamin A/C were used as cytosolic and nuclear marker, respectively. **(C)** Subcellular localization of NFE2L1616–GFP in HEK293 cells. HEK293 cells transfected with NFE2L1616–GFP were fixed and permeabilized, and then visualized with GFP fluorescence (green coloration, left panel). DAPI was used for nuclear counterstaining (middle panel), and the merged image (right panel) shows nuclear localization of NFE2L1616–GFP. Inset in the left panel shows an overlay of bright-field and green fluorescence channel to demonstrate NFE2L1616–GFP localization in the nuclei of cells. Images were taken at × 40 magnification. **(D)** Endogenous NFE2L1616 localizes to the nucleus. Whole cell lysate (W), cytosolic fraction (C), and nuclear fraction (N) from HEK293 and** (E)** ARPE19 were analyzed by SDS-PAGE and immunoblotted with anti-NFE2L1-616 (top panel) and 12936-1-AP (non-isoform specific NFE2L1 antibody, second panel). HEK293 cells transfected with V5-tagged NFE2L1-616 was used as a control for anti-NFE2L1-616 and 12936-1-AP antibodies. Lamin A/C and alpha tubulin were used as a nuclear and cytoplasmic marker, respectively.

To confirm expression of NFE2L1-616 at the protein level, specific rabbit antisera were generated and used for western blotting. Peptides corresponding to the unique amino-terminal sequence deduced from the NFE2L1-616 cDNA were synthesized and used for immunization. Antisera obtained were affinity purified and specificity was examined by western blotting HEK293 cells transfected with an expression vector for NFE2L1-616 with a C-terminal V5-epitope tag. Western blotting with anti-V5 antibody revealed two distinct bands with molecular mass of approximately 100 kDa (Fig. 4A, top panel). The presence of a faint but higher molecular-mass band suggests that NFE2L1-616 undergoes post-translational modification in cells. No bands were detected with anti-V5 antibody in non-transfected control cells. Using the anti-NFE2L1-616 antibody, two similar size bands were detected in NFE2L1-616-V5 transfected cells (Fig. 4A, second panel). As expected, the recombinant protein was detected using a previously validated commercial antibody (12936-1-AP) reactive against a non-overlapping epitope shared by all NFE2L1 isoforms (Fig. 4A, third panel). Control lysates prepared from non-transfected cells showed faint bands matching in size to V5-tagged NFE2L1-616 proteins that likely represent endogenous proteins (Fig. 4A, third panel). As bands detected by the two antibodies correlated with each other, these results indicate that the anti-NFE2L1-616 and 12936-1-AP antibodies can be used to detect the NFE2L1-616 isoform.

Given that NFE2L1-616 lacks the N-terminal sequence found on NFE2L1-772 and NFE2L1-742 that targets these isoforms to the endoplasmic reticulum, we hypothesize that NFE2L1-616 is constitutively nuclear. In HEK293 cells expressing V5-tagged NFE2L1-616, anti-V5 immunoblotting showed that the protein was found in the nuclear fraction of cells (Fig. 4B). Faint signal detected in the cytosolic fraction was likely caused by contamination with nuclear proteins as shown by presence of the nuclear marker, Lamin-A/C. To confirm the western blot data, localization studies using green-fluorescent (GFP)-tagged protein was done. Location of NFE2L1-616 fused with GFP monitored by microscopy showed green fluorescence was detected in the nucleus (Fig. 4C). These results indicate that NFE2L1-616 is localized to the nucleus.

Next, western blotting was done to determine expression of endogenous NFE2L1-616. Using the anti-NFE2L1-616 antibody, a prominent band of approximately 100 kDa was detected in whole cell lysate and the nuclear fraction of HEK293 cells that co-migrated with the lower NFE2L1-616-V5 protein band (Fig. 4D, top panel). To confirm that the 100 kDa protein detected by the NFE2L1-616 antibody was indeed NFE2L1-616, western blotting with 12936-1-AP antibody, which we anticipate would recognize the NFE2L1-616 protein, was done. Using 12936-1-AP antibody, similar sized band was detected in both whole cell and nuclear lysates, and as expected, this antibody recognized the V5-tagged NFE2L1-616 in transfected control cells (Fig. 4D, second panel). We also investigated expression of endogenous NFE2L1-616 proteins in ARPE19 cells. Distinct bands lining up with both the top and lower bands of V5-tagged NFE2L1-616 control were detected in whole cell and nuclear lysates (Fig. 4E). The variation in band intensities could be due to differences in post-translational modifications of NFE2L1-616 protein in ARPE19 vs HEK293 cells. Together, these findings indicate that NFE2L1-616 protein is localized to the nucleus and expressed in human cell lines.

### NFE2L1-616 interacts with MAF-G and is an activator of gene transcription

**Figure 5:**
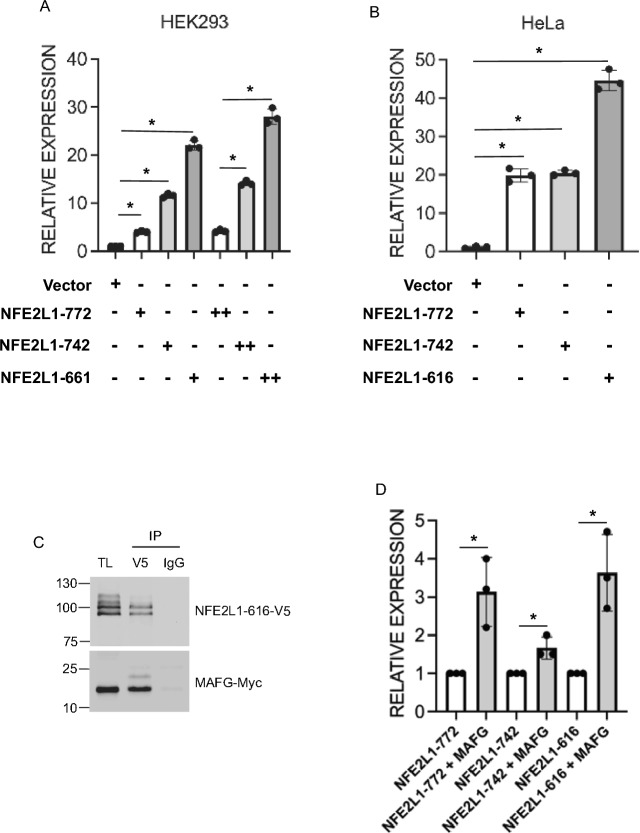
NFE2L1-616 activates the ARE-pathway. **(A)** Transcriptional activity of NFE2L1-616 measured by ARE-driven luciferase reporter assay. HEK293 and **(B)** HeLa cells were co-transfected with empty vector, NFE2L1-772, NFE2L1-742 or NFE2L1-616 expression plasmid along with a reporter construct for NFE2L1 transactivating activity containing copies of the antioxidant responsive element. After 24 h, cells were analyzed for luciferase activity. Luciferase activities were normalized to Renilla luciferase from pRL-TK. Results are expressed relative to luciferase activities observed with empty vector alone. Histograms show the mean of three separate experiments ± SD carried out in triplicates, *P < 0.01. **(C)** Co-immunoprecipitation (Co-IP) showing interaction between NFE2L1-616 and MAFG. HEK293 cells were transfected with NFE2L1-616-V5 and MAFG-Myc. Lysates were prepared 48 h after, and immunoprecipitated with anti-V5 antibody or IgG as control. Immunoprecipitates were then immunoblotted with anti-V5 or anti-Myc antibody. Total lysate (TL) was used as input control. **(D)** Effect of MAFG on ARE-driven luciferase reporter expression. HEK293 cells were co-transfected with ARE-Luciferase reporter, NFE2L1-772, NFE2L1-742 or NFE2L1-616 without or with MAFG expression plasmid. After 24 h, cells were analyzed for luciferase activity. Luciferase activities were normalized to Renilla luciferase from pRL-TK. Results are expressed relative to luciferase activities observed with empty vector alone. Histograms show the mean of three separate experiments ± SD carried out in triplicates, *P < 0.05.

We next determined if NFE2L1-616 can activate gene transcription using a reporter assay. NFE2L1-616-expression plasmid was transiently transfected into HEK293 cells along with the ARE-luciferase reporter plasmid. The activation of luciferase by NFE2L1-616 was dose-dependent on the amount of transfected DNA. At the maximum amount, NFE2L1-616 showed a 30-fold increase over the vector control, while NFE2L1-772 and NFE2L1-742 achieved a 5- and 15-fold activation, respectively. These results indicate that ARE-mediated transcription was strongly stimulated by NFE2L1-616 in HEK293 cells (Fig. 5A). To confirm these results, the stimulatory activities of NFE2L1 proteins were also compared in HeLa cells. Similarly, luciferase activity in HeLa cells was induced by NFE2L1-616, and the activity of NFE2L1-772 and NFE2L1-742 were lower compared to NFE2L1-616 (Fig. 5B). These results indicate that NFE2L1-616 can activate ARE-mediated gene expression.

NFE2L1 proteins form heterodimers with other bZIP transcription factors. Previously, it has been demonstrated that the sMAF family, consisting of three functionally redundant factors, MAFG, MAFF and MAFK, serve as obligate heterodimerization partners of NFE2L1 proteins^25,26^. To test this, interaction between NFE2L1-616 and MAFG was examined. HEK293 cells were co-transfected with V5-tagged NFE2L1-616 and Myc-tagged MAFG, and co-immunoprecipitation analysis showed both proteins interacted with each other (Fig. 5C). These results indicate that NFE2L1-616 can bind MAFG. To determine whether MAFG can also serve as a functional partner, the effect of MAFG on reporter gene transactivation was examined. Co-transfection with MAFG further activated reporter expression by the three isoforms (Fig. 5D).

## Discussion

NFE2L1 transcription factor plays an important role in coordinating cellular stress response by regulating the expression of genes involved in maintaining proteostasis and redox balance in cells^7,27–30^. NFE2L1 has also been implicated in other processes including the differentiation of osteoblast and osteoclast, adipogenesis and adipose function^11–14,31^. There are several isoforms of NFE2L1 reported in the literature. Here, we characterized NFE2L1-616. The NFE2L1-616 protein has a common basic leucine zipper domain of other NFE2L1 proteins but is distinguished by a distinct amino-terminal domain that is generated from an alternate promoter and first exon. In contrast with other long isoforms which are localized to the membrane of the endoplasmic reticulum under steady state condition, NFE2L1-616 is found in the nucleus. Forced expression of NFE2L1-616 promotes ARE reporter activity in HEK293 and HeLa cells. These results suggest that NFE2L1-616 promotes ARE-mediated transcriptional activity.

Recombinant NFE2L1-616 was detected as a protein of approximately 100 kDA using a polyclonal antibody raised against the unique 44-amino acid sequence encoded by the first exon of NFE2L1-616. An endogenous protein of similar size was also detected in cells by the NFE2L1-616-specific antibody, as well as by a previously verified commercial antibody raised against a common region of all NFE2L1 protein isoforms. These results indicate that the NFE2L1-616 transcript is translated into a protein in cells, and a search performed in Ensembl genome database revealed a similar protein in NFE2L1 orthologs in other species suggesting that the NFE2L1-616 isoform is evolutionarily conserved.

Functionality of genomic region upstream of the NFE2L1-616-specific exon-1 to drive luciferase gene expression was tested in HEK293 and HeLa cells, and our results indicate that the second intronic region of NFE2L1 gene can function as an alternative promoter to drive expression of NFE2L1-616. Many human genes have been shown to have alternative promoters, and the use of alternative promoters to drive cell type-specific or stimulus-dependent expression is a common mechanism used in the transcriptional regulation of mammalian genes^32–35^. In this instance however, the NFE2L1-616 promoter is not likely utilized to direct tissue-specific expression as the NFE2L1-616 transcript was detected in various tissues and cell lines examined. The possibility that NFE2L1-616 promoter might direct stimulus-dependent induction of NFE2L1-616 expression will require additional investigation as RT-PCR results showed that NFE2L1-772 and -742 are the predominant isoforms expressed, whereas NFE2L1-616 is generally expressed at lower levels in the cells and tissues that were examined.

The amino acid sequence encoded by the N-terminal exons of NFE2L1-772 and NFE2L1-742 target newly synthesized proteins to the endoplasmic reticulum (ER) membrane^17^. Localization of these long isoforms in the ER provides a mechanism for regulation through a negative-feedback loop. Under normal conditions, these proteins are targeted for destruction by the proteasome as they undergo retrograde membrane transport via the ER-associated degradation machinery^20^. When proteasome capacity is low, NFE2L1-772 and NFE2L1-742 escape degradation and undergo processing by NGLY1 and DDI2 in the cytosol for subsequent translocation to the nucleus to activate expression of downstream genes^18,19^. NFE2L1-616 which is characterized by a distinct N-terminal exon raises the possibility that the subcellular localization of this isoform differs. Indeed, the distribution pattern of endogenous and epitope-tagged recombinant proteins indicates that NFE2L1-616 is localized to the nucleus constitutively. It is interesting to note that NFE2L1-616 was more active in driving luciferase expression compared to other isoforms, which may be consistent with the idea that NFE2L1-616 does not require processing and release from the ER membrane like NFE2L1-772 and NFE2L1-742 isoforms. However, it is not known currently if NFE2L1-616 function is regulated in vivo.

NFE2L1 proteins belong to the CNC subfamily of basic leucine zipper (bZIP) transcription factors function as obligate heterodimers with other bZIP proteins to activate transcription. It was previously demonstrated sMAFs proteins serve as partners of NFE2L1 proteins^25,26^. While our data suggests that NFE2L1-616 can interact and activate reporter expression with the sMAF-bZIP protein, MAFG, identification of endogenous bZIP proteins that dimerizes with NFE2L1-616 requires further experimentation. Previous studies have shown that both NFE2L1-772 and NFE2L1-742 function as positive regulators, mediating basal and stress-induced transcription through the cis-regulatory antioxidant response element (ARE) found in the promoter regions of genes involved in maintaining cellular homeostasis. In our study here, NFE2L1-616 activated transcription from an ARE-containing luciferase reporter gene in HEK293 and HeLa cells suggesting that it can potentially function as a positive regulator of NFE2L1-responsive genes. The existence of multiple isoforms in the same cell may serve as a mechanism to ensure proper expression of NFE2L1-regulated genes. While it is well established that NFE2L1-772 and NFE2L1-742 regulate expression of proteasome genes, the extent to which NFE2L1-616 participates in this pathway in-vivo remains to be elucidated. One possibility is that NFE2L1-616 maintains basal expression of proteasome genes given that it is resident in the nuclear compartment and not subject to ERAD-dependent feedback regulation.

In summary, our data provide evidence for the existence of NFE2L1-616 isoform, which is expressed across a wide range of human cells and tissues. Our data also suggests that NFE2L1-616 promotes transcriptional activity and it may play a role in controlling expression of NFE2L1-regulated genes. Future studies are needed to determine the physiological relevance of NFE2L1-616 and evaluate whether its function is exclusively divergent from other isoforms of NFE2L1 or whether it also includes regulation of cellular stress response.

## Materials and methods

### Reagents

General chemicals for buffers and culture media were purchased from Sigma-Aldrich (St. Louis, MO). Dulbecco’s modified Eagle’s medium (DMEM), Modified Eagle’s Medium (MEM), DMEM/F12, streptomycin, penicillin and fetal bovine serum (FBS) were purchased from ThermoFisher Scientific (Waltham, MA). Bradford protein assay reagent was from BioRad (Hercules, CA). Primary antibodies against Tubulin-A (3873, mouse mAb), Lamin A/C (4C11, mouse mAb), horseradish peroxidase linked anti-rabbit IgG (7074) and anti-mouse IgG (7076) antibodies were from Cell Signaling Technology (Beverly, MA). Rabbit monoclonal antibody against NFE2L1 is from Proteintech (12936-1-AP) which has been validated for specificity using Nrf1 knockout cells.

### Cell lines and cell culture

HEK293, HeLa, HCT116 and ARPE19 cells were from the American Type Culture Collection (Rockville, MD, USA), and they were maintained in Dulbecco’s Modified Eagle’s Medium (DMEM), Modified Eagle’s Medium (MEM), DMEM/F12, respectively supplemented with 10% fetal calf serum, penicillin, and streptomycin and grown at 37 °C in a humidified atmosphere of 5% CO2.

### Expression constructs

The luciferase reporter construct containing a multimerized antioxidant responsive element and Nrf1a-V5 (NFE2L1-742) expression construct were described previously^36^. NFE2L1-616-V5 and NFE2L1-616-GFP were constructed by PCR amplification of NFE2L1-616 cDNA and cloned into pLV-EF1a-LIC-V5 and pcDNA3 GFP LIC (gifts from Larry Gerace, Addgene plasmid # 120248; http://n2t.net/addgene:120248; RRID:Addgene_120248), respectively by ligation independent cloning. The forward and reverse primers for pLV-EF1a-LIC-V5 cloning were 5ʹ-TCGGCCCGCCCATGACCCGCCTGGACCGCʹ and 5ʹ-CCCCCGCCCGGCCCACCCTTTCTCCGGTCCTTTGGʹ. The forward and reverse primers for pcDNA3 GFP LIC cloning were 5ʹ-TACTTCCAATCCAATGCCACCATGACCCGCCTGGACCGCʹ and 5ʹ-CTCCCACTACCAATGCCCTTTCTCCGGTCCTTTGGʹ for pcDNA3 GFP LIC cloning. The resultant constructs were verified by Sanger sequencing. Human MAFG-Myc was purchased from Origene (Rockville, MD).

### Cloning of 5′ RACE products

RNA was isolated from cells, using a Magnetic mRNA Isolation Kit (New England Biolabs) as described by the manufacturer. For 5ʹ RACE analysis, a 5ʹ RACE System (ThermoFisher) was used as described by the manufacturer, with some modifications. Briefly, 1.0 μg of poly(A) + mRNA was reverse transcribed using a NFE2L1-616-specific primer (5ʹ-CAATCAGATCTATGTCCTGAACAG) complementary to sequence located 157 bp downstream of the initiating AUG codon. After RNase treatment and purification, a fifth of the single-stranded cDNA was dC tailed with terminal deoxynucleotidyl transferase. A portion of this reaction was then subjected to amplification using a second NFE2L1-616-specific primer (5ʹ-GTGACCAGCCAGCAAAGAGTT) complementary to sequence located 84 bp downstream of the AUG codon and the 5ʹ RACE abridged anchor primer supplied with the kit (30 cycles of denaturation at 94 °C for 60 s, annealing at 55 °C for 60 s, and extension at 72 °C for 60 s). The primary PCR reaction was diluted 100-fold, and a nested PCR was done on 5 μL of the diluted sample using the 5ʹ RACE abridged universal anchor primer and a third NFE2L1-616-specific primer (5ʹ-CGGGTCATGGTCTGCGAG) that spans the AUG codon (30 cycles of denaturation at 94 °C for 60 s, annealing at 55 °C for 60 s, and extension at 72 °C for 60 s). A distinct PCR product was observed by agarose gel electrophoresis after the secondary PCR, which was cloned into pCR4-TOPO cloning vector (ThermoFisher) and several individual clones were analyzed by Sanger sequencing.

### Alternative NFE2L1-616 promoter-luciferase reporter plasmid

A DNA fragment comprising the region between nucleotide + 152 to − 2417 of the human NFE2L1-616 (numbering relative to the first exon of NFE2L1-616) was generated by PCR amplification and inserted into the KpnI/HindIII sites upstream of the firefly luciferase gene in the pGL4.12 vector (Promega, Madison, WI). Forward and reverse primers were 5ʹ-GATGACGGTACCCCAAGTCAACTTGCTTTGAGC and 5ʹ-GATGACAAGCTTACAACCCCAGTTCCACTCAG. The KpnI or HindIII sites included in these primers are underlined. The resulting construct was verified by Sanger sequencing.

### Luciferase reporter gene assay

Luciferase assays were performed as previously described^37^. Briefly, cells were seeded in 24-well plates, and cells were then transfected 24 h later using Lipofectamine 3000 (ThermoFisher) in quadruplicates with indicated luciferase reporter, effector plasmids, pGL4.74-hRLuc/TK (Promega) internal control plasmids. Empty vector pLV-EFa-LIC-V5 or pEF1aV5 controls were used to keep the amount of transfected plasmid DNA constant. Luciferase activities were measured 24 h after transfection according to the manufacturer’s directions (Promega). The relative luciferase activity was expressed as arbitrary units by normalizing firefly luciferase activity to Renilla luciferase activity. Data represent the average of at least three independent experiments each done in triplicates and error bars represent SD.

### Polyclonal antibody production

Polyclonal antibody production was performed by Pacific Immunology (Ramona, CA). Briefly, a keyhole limpet hemocyanin-conjugated peptides corresponding to the unique 44 amino terminus of NFE2L1-616 (TRLDRSSHSGGGHAKGSRPLNS-Cys, RPLNSLLAGHRVAQPASAPGSRASVQ-Cys), were synthesized and used to immunized two rabbits according to standard protocol. Bleeds from week-10 and 12 were pooled, and affinity purification against the peptides was performed. For purification, sera were diluted 1:1 with PBS (pH7.2) and applied to a peptide affinity column at a flow rate of approximately 0.3 mL/min followed by washing with PBS. Bound antibodies were eluted from the column with 0.2 M glycine (pH 1.85), neutralized with 1 M Tris–HCl (pH 8.5) immediately after collection and concentrated using Amicon Ultra-centrifugal filter columns per manufacturer’s recommendation.

### Quantitative RT-PCR

RNA from human tissues were purchased from Zyagen (San Diego, CA). Total RNA from cells was extracted using Zymo DirectZol RNA Miniprep (Zymo Research, Irvine, CA). Synthesis of cDNA was done using iScript Advanced cDNA Synthesis Kit (Bio-Rad, Hercules, CA). Quantitative RT-PCR was performed using Luna Universal Probe qPCR Master Mix (NEB, Ipswich, MA) in a QuantStudio 3 Real-Time PCR system running QuantStudio Design and Analysis Software (v1.5.1, Applied Biosystems) with the following primers: NFE2L1-772 forward-GGGAGAATGCTGAGTTTC, reverse-GAGACAAGAGGTTGTTTTG, probe-6FAM-CTCTCACTAGGCACTGCTTCTGT-BHQ1; NFE2L1-742 forward-CTTGGATGGCTATGGTATC, reverse-CAGGCATTTACCTCAGTG, probe-6FAM-AGCTTCCCTGCACAGTTTCCA-BHQ1; NFE2L1-616 forward-CTCCCTGAAAGCCAAGAG reverse-CAGCCAGCAAAGAGTTAAG, probe-6FAM-CCTGTCCGGCTTCCCGGCAC-BHQ1; RPLP0 forward-GGCTTTGTGTTCACCAAG, reverse-CACAGTGACTTCACATGG, probe-6FAM-ACCTCACTGAGATCAGGGACATG-BHQ1; ALAS1 forward-GAGACAGATGCTAATGGA, reverse-CTTGCACGTAGATGTTATG, probe-6FAM-TGCTCATTAGTTCATCACAGACTTCT-BHQ1. PCR cycling conditions consist of 95 °C for 10 min and 40 cycles of 95 °C for 10 s and 60 °C for 30 s. Results were calculated using the ΔΔCT method with RPLP0 or ALAS1 as reference genes.

### Western blotting

Cells were lysed in RIPA buffer containing a cocktail of protease inhibitors (ThermoFisher PI78410). Lysates were cleared by centrifugation at 4 °C, 12,000×*g* for 15 min, and protein concentrations measured using Bradford assay. Protein samples were denatured with an equal volume of 2× SDS sample buffer (100 mM Tris, pH 6.8, 25% glycerol, 2% SDS, 0.01% bromphenol blue, 10% 2-mercaptoethanol), and the mixture boiled for 5 min. Proteins were electrophoresed on SDS-PAGE gels and transferred onto nitrocellulose membranes. Membranes were then blocked in 5% skim milk in TBS-T (150 mM NaCl, 50 mM Tris–HCl pH 8.0, and 0.05% Tween 20) at room temperature for 1 h, and then incubated with the indicated primary antibodies at 1:1000 dilution (unless otherwise indicated) overnight at 4 °C followed by incubation with 1:2000 dilution of horseradish peroxidase-conjugated secondary anti-rabbit, or anti-mouse antibody. Antibody-antigen complexes on the blots were detected using a chemiluminescent detection system. Antibody reactive against non-overlapping epitopes shared by all NFE2L1 isoforms is from Proteintech (12936-1-AP), and it has been previously validated for specificity using Nrf1 knockout cells or mouse tissues^38^. Cell Fractionation kit (Cell Signaling #9038) was used according to manufacturer’s instruction for the preparation of nuclear and cytoplasmic fractions, and Lamin-A/C and Tubulin-A were detected as nuclear and cytoplasmic markers, respectively.

### Immunoprecipitation assays

Transfected cells were lysed in RIPA buffer containing protease inhibitors as described above. Lysates were then centrifuged for 15 min at 10,000×*g*, and an aliquot was retained to measure the total protein content, and the remainder was subjected to pre-clearing with protein-G Sepharose beads by incubation at 4 °C for 1 h. After centrifugation, the supernatants were incubated at 4 °C with 2–5 μg of primary antibody, and controls with no antibody were also included. After overnight incubation, protein-G Sepharose beads were added and incubated at 4 °C for 1 h. Beads were then collected by brief centrifugation and washed extensively with RIPA buffer. The bound proteins were then dissociated from the beads by heating at 75 °C for 5 min, resolved by SDS-PAGE, and analyzed by western blotting as described above.

### Fluorescence microscopy and subcellular fractionation

Cells were seeded onto Nunc Lab Tek II glass chamber slides and transfected with NFE2L1-616-GFP. After 48 h, cells were counterstained with 4ʹ,6-diamidino-2-phenylindole (DAPI; Biotium) at a final concentration of 1 μg/mL for 30 min. Cells were then examined with a Nikon epifluorescent microscope using a FITC filter set for GFP, and UV for blue fluorescence for DAPI. Images were acquired using a 63× objective lens with a Spot RT3 digital camera and Adobe Photoshop CS was used to layer the captured images.

### Statistical analysis

All data were expressed as the means ± standard deviation (SD) of three or more individual experiments. Statistical analysis was performed using Prism software (GraphPad, La Jolla, CA). Differences in mean values between the two groups were tested by the Student’s t-test. *P-values < 0.05 were considered statistically significant.

## Data Availability

All data generated or analyzed during this study are included in this published article.
